# *Physalis peruviana* L. Pulp Prevents Liver Inflammation and Insulin Resistance in Skeletal Muscles of Diet-Induced Obese Mice

**DOI:** 10.3390/nu12030700

**Published:** 2020-03-05

**Authors:** Francisco Pino-de la Fuente, Diego Nocetti, Camila Sacristán, Paulina Ruiz, Julia Guerrero, Gonzalo Jorquera, Ernesto Uribe, José Luis Bucarey, Alejandra Espinosa, Luis Puente

**Affiliations:** 1 Departamento de Tecnología Médica, Facultad de Medicina, Universidad de Chile, Santiago 8380453, Chile; pinodelafuente.francisco@gmail.com (F.P.-d.l.F.); c_sacristan@ug.uchile.cl (C.S.); paruiz@uchile.cl (P.R.); ernesto.uribe@live.com (E.U.); bespinosa@med.uchile.cl (A.E.); 2 Programa de Doctorado en Ciencias Médicas, Universidad de La Frontera, Temuco 4811230, Chile; diego.nocetti.g@uta.cl; 3 Departamento de Tecnología Médica, Universidad de Tarapacá, Arica 1010069, Chile; 4 Programa de Fisiología, Instituto de Ciencias Biomédicas, Facultad de Medicina, Universidad de Chile, Santiago 8380453, Chile; jguerrero@med.uchile.cl; 5 Departamento de Medicina Interna, Hospital Clínico—Universidad de Chile, Santiago 8380456, Chile; 6 Centro de Neurobiología y Fisiopatología Integrativa, Instituto de Fisiología, Facultad de Ciencias, Universidad de Valparaíso, Valparaíso 2391415, Chile; gonzalo.jorquera@uv.cl; 7 Escuela de Medicina, Campus San Felipe, Universidad de Valparaíso, San Felipe 2340000, Chile; jose.bucarey@uv.cl; 8 Departamento de Ciencias de los Alimentos, Facultad de Ciencias Químicas y Farmacéuticas, Universidad de Chile, Santiago 8380494, Chile

**Keywords:** lipid droplets, insulin resistance, Cape gooseberry

## Abstract

A chronic high-fat diet (HFD) produces obesity, leading to pathological consequences in the liver and skeletal muscle. The fat in the liver leads to accumulation of a large number of intrahepatic lipid droplets (LD), which are susceptible to oxidation. Obesity also affects skeletal muscle, increasing LD and producing insulin signaling impairment. *Physalis peruviana* L. (PP) (Solanaceae) is rich in peruvioses and has high antioxidant activity. We assessed the ability of PP to enhance insulin-dependent glucose uptake in skeletal muscle and the capacity to prevent both inflammation and lipoperoxidation in the liver of diet-induced obese mice. Male C57BL/6J mice were divided into groups and fed for eight weeks: control diet (C; 10% fat, 20% protein, 70% carbohydrates); C + PP (300 mg/kg/day); HFD (60% fat, 20% protein, 20% carbohydrates); and HFD + PP. Results suggest that PP reduces the intracellular lipoperoxidation level and the size of LD in both isolated hepatocytes and skeletal muscle fibers. PP also promotes insulin-dependent skeletal muscle glucose uptake. In conclusion, daily consumption of 300 mg/kg of fresh pulp of PP could be a novel strategy to prevent the hepatic lipoperoxidation and insulin resistance induced by obesity.

## 1. Introduction

Obesity is the result of the imbalance between calorie intake and expenditure, resulting in the accumulation of adipose tissue, which is potentiated by physical inactivity [1,2]. Integrated metabolism is promoted by the coordinated action of insulin in the liver, adipose tissue, and skeletal muscle. The interplay between the functions of these organs contributes to systemic glucose regulation. Fasting glucose is maintained by glucose release from the liver by glycogenolysis and gluconeogenesis. Under normal diet, insulin secreted by the pancreas regulates physiological glucose concentration, acting on skeletal muscle and adipose tissue to favor glucose uptake in an insulin-dependent way [3,4]. With over-feeding, metabolic alterations in different tissues disturb glucose homeostasis, triggering a systemic insulin resistance (IR) condition [5]. Liver-specific metabolic abnormalities are present in IR-associated obesity, such as Non-Alcoholic Fatty Liver Disease (NAFLD) and steatohepatitis [6]. IR in the liver is a paradoxical process, referred to as “selective hepatic insulin resistance,” in which both glucose production and lipid storage are increased, altering the normal function of the liver by the increase of gluconeogenesis and *de novo* lipogenesis [5]. Lipid β-oxidation is also impaired, favoring the accumulation of lipid droplets (LDs), which in turn are the substrate for lipid peroxidation processes. The high presence of oxidized lipid adducts contributes to “lipotoxicity,” which worsens the IR state and promotes hepatocyte damage by oxidative stress [7]. Obesity is characterized by the increase of adipose tissue, which presents abnormalities due to the hypertrophied and poorly oxygenated adipocytes. This dysfunctional adipose tissue is prone to secrete inflammatory cytokines such as TNFα, IL-6, and monocyte chemoattractant protein-1 (MCP1), among others. In obesity, hypertrophic adipocytes secrete pro-inflammatory adipokines, which recruit macrophages, amplifying the inflammatory signals [8]. Hepatic tissue also recruits macrophages in response to the increased free fatty acids (FFAs) overflow. Both local and systemic inflammation levels promote the progression of NAFLD [7,8].

It is well known that inflammation plays a critical role in the IR state of skeletal muscle. FFAs excess is capable of increasing expression of pro-inflammatory cytokines in macrophages, activating NF-κB and JNK pathways, both associated with abnormal insulin signaling, triggering the impairment of insulin-dependent GLUT4 translocation in skeletal muscle [9].

Some anti-obesogenic therapies based on fruit administration are thought to be useful to decrease the metabolic consequences associated with overweight and obesity. Dietary interventions in which nutraceutical products have been incorporated prevent some of the metabolic alterations related to obesity induced by diet [10,11]. These fruits share as common characteristics their high content of polyphenols, given by phenolic components which have greater antioxidant and anti-inflammatory effects [12,13]. For example, the chronic administration of green tea polyphenols reduces lipid infiltration, and both inflammatory and oxidative stress markers in the liver of HFD-fed rats [14]; blueberry extract also protects NAFLD-associated hepatocyte damage, inhibiting inflammatory and oxidant signals through the suppression of NF-ĸB activation [15]. An extract from strawberry and blueberries ameliorates the metabolic alterations induced by obesity such as IR and inflammation [16]. *Physalis peruviana* L. (PP) is distributed across South America; it is an abundant source of phenolic components, like coumarin, kaempferol, quercetin, and ascorbic acid, which have several biological activities including the prevention of some metabolic abnormalities associated with obesity [17]. Some of the beneficial effects of PP are described in different reports; PP extract reduced the formation of reactive oxygen species (ROS) and preserved mitochondrial membrane potential in astrocytes [18]; it attenuated airway inflammation associated with chronic obstructive pulmonary disease by inhibiting ROS production and activating antioxidant defense of the epithelial cells [19]. In the liver, the PP juice reduced CCl_4_—associated with liver injury, decreasing the lipoperoxidation level [20].

The intracellular mechanism by which PP produce some of its effects on specific tissues affected by HFD-induced obesity has not yet been studied. The purpose of our study is to assess the effect of fresh PP pulp in the prevention of metabolic alterations associated with obesity in the liver and skeletal muscle in diet-induced obese mice. The results show that the PP pulp has a hepatoprotective effect, decreasing pro-inflammatory markers. PP also improves IR in skeletal muscle, favoring insulin-dependent glucose uptake, and reduces the lipoperoxidation level.

## 2. Materials and Methods

### 2.1. Animals

Weaned healthy males (C57/BL6, four weeks old, 20 ± 2 g) were obtained from the Public Health Institute, Santiago, Chile. All mice were housed in a temperature-controlled room with a 12 h:12 h light/dark cycle and were given access to their specific diet and water *ad libitum*. Thirty-two C57BL/6 male mice were randomly divided into four groups with equal numbers of animals (n = 8) and fed for 8 weeks: control diet (C; 10% fat, 20% protein, 70% carbohydrates, D12450B, Research Diets, New Brunswick, NJ, USA, n); C + PP (300 mg/kg/day for 8 weeks); HFD (60% fat, 20% protein 20% carbohydrates, D12492, Research Diets, New Brunswick, NJ, USA); or HFD + PP. A non-toxic PP dose of 300 mg/kg/day was chosen based on previous reports [21]. All animals were cared for according to the Ethics Committee of the Facultad de Química y Farmacia, Universidad de Chile (CBE 2017-10). At the end of the experimental period, mice were euthanized according to the protocol; tissues (hepatic and skeletal muscle) and blood samples were collected on the same day.

### 2.2. Fruits and Mice Supplementation

PP fruits were purchased from the local producer Hortifrut, Santiago, Chile. The samples were selected to provide a homogenous group, based on the date of harvest, color, size, and freshness according to visual analysis. While still fresh, the fruits were pressed with peel and seeds and homogenized in a blender to obtain pulp (Oster Xpert). Pulp was aliquoted and stored at −80 °C in 1 mL vials. One vial was unfrozen every day for oral animal supplementation, using oral gavage with a polyethylene tube (ADAMS, INC. New York, NY, USA) attached to a 1 cc syringe (Becton Dickinson, Franklin Lakes, NJ, USA).

### 2.3. Measurements of Serum Parameters

Serum was collected after blood centrifugation at 4000 g at 4 °C for 15 min and stored at −20 °C. Serum insulin concentrations were determined by a commercially available immunoassay specific for mice (Mercodia, Uppsala, Sweden). Biochemical parameters were measured by dry chemistry technology (SPOTCHEM™ EZ, Minneapolis, USA). The HOMA-IR (homeostasis model assessment of insulin resistance) was calculated as follows: HOMA-IR = [fasting glucose (mg/dL) × fasting insulin (µU/mL)]/405.

### 2.4. Intraperitoneal Glucose Tolerance Test (IpGTT)

An intraperitoneal glucose tolerance test (IpGTT) was performed one week before euthanasia, after 4 h of fasting by the administration of a 2 g/kg intraperitoneal glucose solution, recording glycemia at 0, 15, 30, 60, 90 and 120 min after injection, using tail blood samples. Blood glucose concentrations were measured in a commercial glucometer (Johnson and Johnson, New Brunswick, NJ, USA).

### 2.5. Skeletal Fiber Culture

The skeletal fiber culture was described previously [22]. Briefly, isolated fibers from Flexor digitorium brevis (FDB) muscle were isolated by collagenase type 2 (Worthington, Lakewood, NJ, USA) digestion for 60 min at 37 °C. Then the muscle was mechanically dissociated using fire-polished Pasteur pipettes. Isolated fibers were seeded in Matrigel-coated coverslips. Dulbecco’s modified Eagle’s medium (Invitrogen, Carlsbad, CA, USA) supplemented with 10% horse serum (Invitrogen) was used to maintain the culture for the next 12 h. 

### 2.6. Glucose Uptake Assay

Skeletal muscle fibers were obtained from mice as described before [22], washed with Krebs-Ringer buffer (in mmol/L: 20 HEPES-Tris (pH 7.4), 118 NaCl, 4.7 KCl, 3 CaCl_2_, 1.2 MgCl_2_ and pre-incubated with 10 nM insulin for 20 min. Then fibers were exposed to 2-[N-(7-nitrobenz-2-oxa-1,3-diazol-4-yl)amino]-2-deoxy-d-glucose (2-NBDG, Thermo Fisher Scientific, Waltham, MA, USA) (300 μmol/L) for 20 min and rinsed with Krebs buffer. Individual skeletal fibers were visualized using a confocal Nikon Spectral C2^+^ microscope (Nikon, Tokyo, Japan) after 488 nm excitation. Fluorescence intensity in both channels was quantified by FIJI (National Institutes of Health, Bethesda, MD, USA).

### 2.7. Quantitative PCR

Total RNA was obtained from liver samples employing homogenization beads and Trizol reagent (Invitrogen, Corp., Carlsbad, CA, USA) according to the manufacturer’s protocol. DNase treatment was done with TURBO DNA-free™ Kit (Thermo Fisher Scientific, Waltham, MA, USA) following the manufacturer’s instructions. cDNA was prepared from 1 μg of RNA, using the SuperScript II enzyme (Thermo Fisher Scientific, Waltham, MA, USA), according to manufacturer’s protocol. Real-time PCR was performed using Stratagene M × 3000P (Stratagene, La Jolla, CA, USA) using the Brilliant III Ultra-Fast QPCR & QRT-PCR Master Mix amplification kit (Agilent Technologies, Santa Clara, CA, USA). The primers used were: TNF-α: 5′-CTGAACTTCGGGGTGATCGG-3′ (sense), 5′-GGCTTGTCACTCGAATTTTGAGA-3′ (antisense); IL-1β: 5′-GCAACTGTTCCTGAACTCAACT-3′ (sense), 5′-ATCTTTTGGGGTCCGTCAACT-3′ (antisense); IL-6: 5′-CCAATTTCCAATGCTCTCCT-3′ (sense), 5′-ACCACAGTGAGGAATGTCCA-3′ (antisense); TLR4: 5′-ATGGCATGGCTTACACCACC-3′ (sense), 5′-GAGGCCAATTTTGTCTCCACA-3′ (antisense); P0: 5′-CTCCAAGCAGATGCAGCAGA-3′ (sense), 5′-ATAGCCTTGCGCATCATGGT-3′ (antisense). All primers used presented optimal amplification efficiency (between 90% and 110%). PCR amplification of the housekeeping gene P0 was performed as a control. Thermocycling conditions were as follows: 95 °C for 3 min and 40 cycles of 95 °C for 10 s, 60 °C for 20 s. Expression values were normalized to P0 and are reported in units of 2-ΔΔCT ± SD as described [23]. CT values were determined by MXPro software when fluorescence was 25% greater than the background. PCR products were verified by melting-curve analysis.

### 2.8. Lipoperoxidation Labeling

To measure the lipoperoxidation (LPO) level and analyze lipid droplets, 4,4-difluoro-5-(4-phenyl-1,3-butadienyl)-4-bora-3a,4a-diaza-s-indacene-3-undecanoic acid (BODIPY^®^ 581/591 C11) (Invitrogen, Carlsbad, CA, USA) was used. This dye shifts from red to green upon oxidation. Freshly isolated hepatocytes or isolated cultured skeletal fibers were incubated with BODIPY 581/591 for 60 min at room temperature. Green and red fluorescence signals were captured by confocal microscopy (Nikon Spectral C2^+^ microscope, Tokyo, Japan). An acquisition protocol using simultaneously double wavelength excitation (laser lines 488 and 568 nm) was used. Fluorescence intensity in both channels was quantified by FIJI (NIH, Bethesda, MD, USA). (National Institutes of Health, Bethesda, MD, USA).

### 2.9. Statistics

Data are presented as mean ± SEM. Significant differences between and within multiple groups were examined using 1-way ANOVA for repeated measures, followed by Newman-Keuls multiple comparison test; *p* < 0.05 was considered statistically significant (IC 95%). All statistical analyses were performed using GraphPad Prism 5.

## 3. Results

### 3.1. Weights and Metabolic Parameters in HFD-Fed Mice Supplemented with PP

After eight weeks of HFD feeding, mice showed an obese phenotype given by total weigh greater than the control condition and increase of visceral fat. Supplementation with PP prevents visceral fat accumulation but does not influence total weight (Table 1).

HFD-fed mice showed higher glycemia levels than control groups, and PP administration prevented fasting glycemia increase induced by HFD. Triacylglyceride levels were not different among the groups, but total cholesterol level was higher in HFD-fed mice compared to the control group. PP treatment had no effect on decreasing cholesterol levels in obese mice. To assess a possible liver injury associated with HFD feeding, alanine aminotransferase (ALT) was measured, but no differences were found among the four groups.

**Figure 1:**
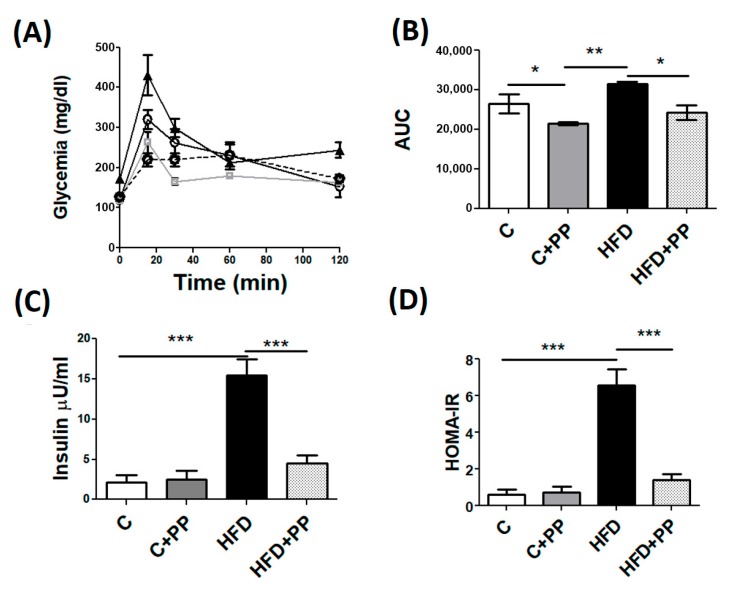
Glucose homeostasis in mice treated with *Physalis peruviana* L. (PP). (**A**) Intraperitoneal glucose tolerance test (ipGTT) was performed after 4 h fasting by the administration of 2 g/kg glucose. (**B**) The area under the curve from A. (**C**) Fasting serum insulin concentration. (**D**) HOMA-IR was calculated [fasting glucose (mg/dL) × fasting insulin (µU/mL)]/405. Data are presented as mean ± SEM, * *p* < 0.05; ** *p* < 0.01; *** *p* < 0.005, (*n* = 8 animals per group). C: control diet; C + PP: control diet supplemented with PP; HFD: high-fat diet; HFD + PP: high-fat diet supplemented with PP.

To evaluate glucose homeostasis, an ipGTT was performed one week previous to euthanasia, which showed that HFD feeding produced glucose intolerance after eight weeks (Figure 1A,B), and PP supplementation prevented this alteration in the HDF-fed group. PP also improved glucose tolerance in the control group. Both blood insulin measurement and the insulin resistance approximation using HOMA-IR (Figure 1C,D) showed that HFD feeding caused alterations in glucose homeostasis. HFD mice supplemented with PP pulp significantly decreased serum insulin (4.5 ± 0.9 μU/mL) compared to the HFD group (15.5 ± 1.9 μU/mL). The decrease of both insulin concentration and HOMA-IR by the administration of PP pulp in HFD-fed mice indicates that the fruit pulp effectively improves systemic insulin sensitivity.

### 3.2. Hepatic Obesity-Induced Lipoperoxidation is Prevented by PP Supplementation

**Figure 2:**
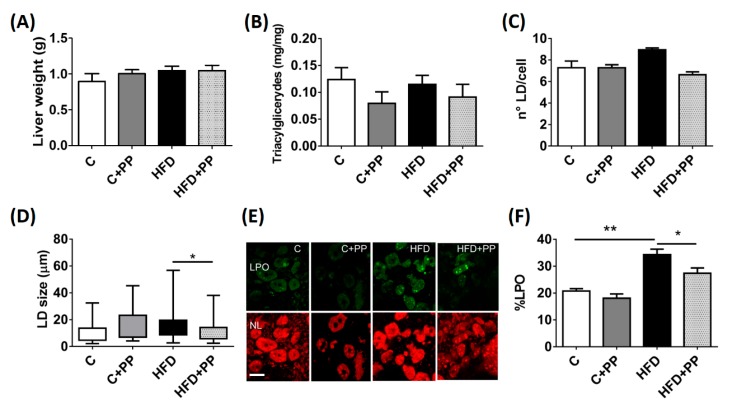
*Physalis peruviana* L. prevents lipid droplet (LD) oxidation in the liver from high-fat fed mice. (**A**) Liver weight. (**B**) Liver triacylglyceride content. (**C**) Number of LDs per hepatocyte. (**D**) The graph shows the median LDs diameter from isolated hepatocytes of each group. (**E**) Representative field showing the lipoperoxidation label (LPO) in green and neutral lipid stain (NL) in red for isolated hepatocytes after 60 min incubation with BODIPY C11. Images were acquired by confocal microscopy (600× magnification, scale bar = 50 µm). (**F**) The graph represents the %LPO after quantification of the fluorescent intensity of each LD using the FIJI program. C: control diet; C + PP: control diet supplemented with PP; HFD: high-fat diet; HFD + PP: high-fat diet supplemented with PP. Data are presented as mean ± SEM (n = 8 animals per group); * = *p* < 0.05; ** = *p* < 0.01.

Considering that HFD induces lipid accumulation in the liver, we focused on evaluating whether PP is protective when faced with the fat increase in isolated hepatocytes and whole liver tissue. The effect of PP administration on liver fat, weight, and triacylglyceride content was assessed in the four groups. No differences among groups were found (Figure 2A,B). These results showed that restrictive HFD does not affect liver lipid content over eight weeks. PP administration also did not affect liver lipid content in the control or in the HFD-fed group. The number of intracellular LDs did not increase significantly in HFD groups (Figure 2C), but the size of LDs was greater than those present in the hepatocytes from the control group. The larger size of LDs in HFD-fed mice was not present in the group supplemented with PP (Figure 2D). LDs are susceptible to suffer lipoperoxidation of the membrane. A specific probe to sense the lipoperoxidation (LPO) level was used in isolated hepatocytes of the studied groups. HFD induced an increase of the LPO percentage with respect to the control group (19 ± 2 vs. 34 ± 3), as can be appreciated in the green label observed in the freshly isolated hepatocytes (Figure 2E,F). Hepatocytes from HFD supplemented with PP reached a lower percentage of LPO (28 ± 2). This result suggests a protective role of the components of PP against the oxidative stress caused by chronic HFD in the liver.

### 3.3. Pro-Inflammatory Markers in the Liver of HFD-Induced Obese Mice are Prevented with PP Supplementation

**Figure 3:**
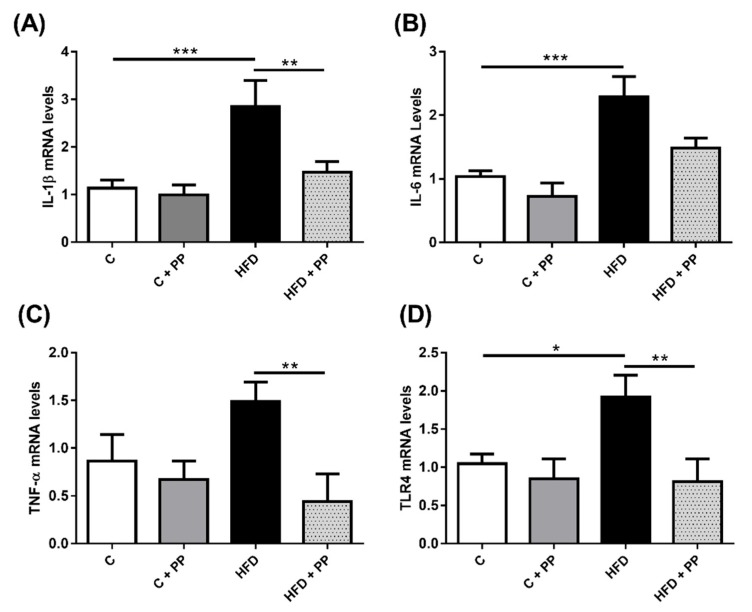
*Physalis peruviana* L. (PP) prevents pro-inflammatory markers expression in liver from mice fed with HFD. (**A**) Interleukin-1β (IL-1β), (**B**) Interleukin-6 (IL-6), (**C**) Tumor necrosis factor-α (TNF-α), and (**D**) Toll-like receptor 4 (TLR4) mRNA levels in liver samples were analyzed through qPCR. Liver sections obtained from mice fed with the high-fat diet (HFD) showed a high expression of pro-inflammatory markers associated with NAFLD compared to samples obtained from mice fed with the control diet (C) and with the control diet supplemented with PP (C + PP). PP extracts given to high-fat fed mice (HFD + PP) prevented pro-inflammatory marker expression in the liver. Data were normalized to fold change with respect to control conditions. P0 expression was used as housekeeping. Data are presented as mean ± SEM (*n* = 4 liver per group analyzed). * *p* < 0.05; ** *p* < 0.01; *** = *p* < 0.005.

TNF-α, IL-6, and IL-1β are pro-inflammatory cytokines involved in the development of inflammation in NAFLD; they have been associated with the disease progression [24,25,26]. Toll-like receptor 4 (TLR4) has also been associated with inflammatory activation the in the liver of obese patients and has been related to nonalcoholic steatosis and steatohepatitis development [26,27]; the increase of LPO level should be an inflammatory stimulus in the liver in this condition. To assess whether in this diet-induced obesity model there are elevated values of liver inflammatory markers and PP effect, cytokines, and TLR4 gene expression were measured in liver homogenates. TNF-α, IL-6, IL-1β, and TLR4 mRNA levels in the HFD-fed mice group were significantly increased compared to mice in the control group. PP significantly decreased cytokines and TLR4 mRNA levels in liver samples of HFD-fed animals, while in the control group it had no effect (Figure 3).

### 3.4. PP Improves Insulin-Dependent Glucose Uptake and Reduces Lipoperoxidation Level in Skeletal Muscle Fibers from HFD-Fed Mice

**Figure 4:**
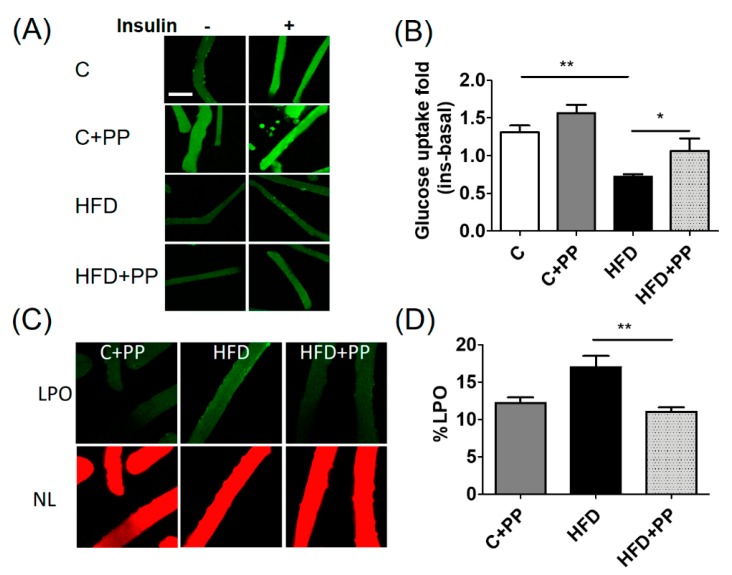
Insulin-dependent glucose uptake and lipoperoxidation levels in the adult skeletal muscle fibers from high-fat fed mice. (**A**) Representative images from cultured isolated skeletal muscle fiber both in basal condition and 20 min after insulin addition (magnification 400×, scale bar = 200 µm) (**B**) Quantification of fluorescence intensity of the 2-NBDG probe, (insulin-basal, 10–20 fibers per condition, *n* = 8 animals per group). (**C**) A panel of images showing the lipoperoxidation label (LPO) in green and neutral lipid stain (NL) in red from isolated skeletal fibers after 60 min incubation with BODIPY C11. Images were acquired by confocal microscopy (400× magnification). (**D**) The graph represents the %LPO after the quantification of the fluorescent intensity of whole fiber, using the FIJI program. C: control diet; C + PP: control diet supplemented with PP; HFD: high-fat diet; HFD + PP: high-fat diet supplemented with PP. Data are presented as mean ± SEM. * = *p* < 0.05; ** = *p* < 0.01.

The results of insulin-dependent glucose uptake in cultured isolated skeletal fibers showed that glucose uptake was 45% less in HFD mice than the control group. The PP + HFD group reached 78% of the glucose uptake shown by the control condition (Figure 4A,B). These results suggest that PP treatment prevents the loss of insulin sensitivity in skeletal muscle. Besides, LPO levels were measured in skeletal muscle, and the %LPO was 70% greater in HFD condition than in the control condition. The PP + HFD group showed 25% more LPO label than the control group. These results suggest that PP prevents the increase of LPO induced by obesity in HFD-fed mice.

## 4. Discussion

Obesity is caused by an imbalance between lipolysis and lipogenesis, where the latter is the predominant condition. There are several mechanisms causing the increase of adipose mass, including high caloric diet consumption, reduced physical activity, and aging [27,28]. Obesity is a leading cause of NAFLD driving an aberrant accumulation of lipids in the liver, which produces inflammatory signals that recruit immune cells [8,23].

The net effect of the accumulation of triacylglycerides, as well as other lipid intermediaries such as diacylglycerol, is to inhibit insulin signaling in its target tissues [29]. The pro-inflammatory scenario also affects essential tissues associated with metabolic homeostasis; skeletal muscle, which is the primary regulator of postprandial glycemia, can be altered by lipid intermediaries, pro-inflammatory cytokines and ROS, promoting an IR status [21,30]. Several nutritional strategies are being proposed to achieve the prevention of metabolic abnormalities, including diet supplementation of foods with a high content of bioactive phytochemicals. Polyphenols are present in a wide variety of fruits such as apples, grapes, and raspberry, producing benefits through decreasing fat accumulation in mice fed with a HFD [29,30,31]. This study assessed the effect of PP pulp administration on tissue-specific alteration induced by obesity, focusing on the inflammatory and redox disequilibrium in both skeletal muscle and liver. This is the first study that has explored the specific intracellular effects of PP pulp in the prevention of metabolic alterations and increasing insulin sensitivity in both skeletal and hepatic tissue.

Our results showed that PP pulp has a protective effect against systemic abnormalities such as the fasting increase of both glycemia and insulin, indicating that PP treatment prevents the loss of glucose management associated with obesity. The hypoglycemic effect was reported before [29], showing that PP consumption is a strategy for complementing effective anti-diabetes treatment. Here we have shown that PP also can regulate insulin levels and that the mechanism involved is probably dependent on skeletal muscle protection.

Several studies have shown that there is a protective role of PP due to its anti-inflammatory properties; this effect has been associated with different parts of the PP plant [25,26]. PP calyces have already been reported to decrease nitric oxide and prostaglandin E2 in injection-induced λ-carrageenan paw edema in mice [29]. Inflammatory cytokines and chemokines were markedly decreased with PP leaf extract administration in a lipopolysaccharide (LPS)-induced inflammation airway model, in which the influx of inflammatory cells and the expression of monocyte chemoattractant protein-1 (MCP-1) in the inflamed lungs was reverted [19]. However, few reports have shown that PP fruits have anti-inflammatory effects [20]; our results add to the growing body of evidence showing that PP pulp decreases pro-inflammatory markers in the liver. They also show that pro-inflammatory cytokines elevated by a HFD diet, as previously was reported, [29] did not increase under the effect of PP supplementation.

We also report that although PP pulp supplementation did not decrease the total lipid content in the liver of HFD-fed mice, the LDs were smaller than LDs from the HFD-fed group. Recently the importance of LD size was reported; regular exercise in HFD-fed mice induced an increase in the number of LDs, but the area of LD was reduced, which was associated with less liver damage [30].

Considering that skeletal muscle is one of the main tissues involved in the systemic metabolism after meal ingestion [3], it was crucial to study the effect of PP treatment in glucose management by this tissue. Here we report for the first time that PP could improve insulin-dependent glucose uptake in isolated skeletal muscle fibers from HFD-fed mice. It has been suggested that hypoglycemic effects are apparently due to the high amount of sucrose esters, called peruvioses, through the inhibitory effect on the α-amylase enzyme, but no reports have shown the effects of peruvioses in vitro in skeletal muscle [17,28], which could be an interesting future perspective considering that skeletal muscle is an essential target to manage glucose homeostasis. The best candidates among the components present in PP to improve glucose uptake are withanolides [17,31]. Gorelick et al. reported an increase in insulin-dependent glucose uptake in skeletal muscle cell line C2C12 after the treatment with a *W. somnifera* extract rich in withanolides [32].

One of the mechanisms involved in the onset of obesity-dependent insulin resistance is the increase of intracellular ROS. We have previous experience in an HFD-model in which obesity and insulin resistance is developed in two months, accompanied by an intracellular oxidative environment in skeletal muscle [22]. Our current data show that HFD induces an increase in LPO levels in both isolated hepatocytes and isolated skeletal muscle cells. PP treatment prevented the increase of LPO levels in both cell types. Avoiding an LPO increase is a basic way to avoid the consequences of insulin resistance because excessive ROS production potentiates the insulin signal impairment induced by obesity.

## 5. Conclusions

In conclusion, the daily consumption of 300 mg/kg of fresh pulp of PP prevented the increase of pro-inflammatory markers and LPO levels in the liver and LPO in skeletal muscle of HFD-fed mice. Fruit supplementation prevents the abnormalities in glucose homeostasis by improving glucose uptake in adult skeletal muscle.

## Figures and Tables

**Table 1:** Effect of dietary *Physalis peruviana* L. (PP) supplementation on weight and serum biomarkers.

	C (a)	C + PP (b)	HFD (c)	HFD + PP (d)
Total Weight (g)	22.7 ± 1.1 ** (d) *** (c)	26.8 ± 0.8 ** (c, d)	38.6 ± 1.6 ** (b) *** (a)	35.8 ± 3.4 ** (a, b)
Visceral Fat Weight (mg)	147.0 ± 0.5 ** (d)	156.0 ± 1.6 *** (b)	407.0 ± 7.0 ** (a), *** (b)	214.0 ± 3.1 ** (a, c)
Fasting glycemia (mg/dL)	140.6 ± 22.4 ** (c, d)	127.3 ± 3.9 *** (c), ** (d)	190.5 ± 7.2 ** (a), *** (b)	168.1 ± 10.7 ** (a, c), *** (b)
Triacylglycerides (mg/dL)	66.7 ± 8.9	46.0 ± 2.4	52.2 ± 4.1	55.3 ± 2.9
Total cholesterol (mg/dL)	94.5 ± 3.2	89.2 ± 3.8	135.2 ± 5.7 ** (a, b)	130.7 ± 9.6 ** (a, b)
ALT (UI/l)	53.0 ± 5.3	62.0 ± 12.3	52.0 ± 6.5	67.0 ± 7.4

C: control diet (a); C + PP: control diet supplemented with *Physalis peruviana* (PP) (b); HFD: high-fat diet (c); HFD + PP: high-fat diet supplemented with PP (d). Supplementation consisted in 300 mg/kg/day of PP pulp, over 8 weeks. All measurements were made after 4 h of fasting. Data are presented as mean ± SEM (*n* = 8 animals per group). ** *p* < 0.01; *** *p* < 0.005.
